# Spatial and temporal distribution of foot-and-mouth disease in four districts situated along the Uganda–Tanzania border: Implications for cross-border efforts in disease control

**DOI:** 10.4102/ojvr.v85i1.1528

**Published:** 2018-08-27

**Authors:** Susan D. Kerfua, Gabriel Shirima, Lughano Kusiluka, Chrisostome Ayebazibwe, Robert Mwebe, Sarah Cleaveland, Daniel Haydon

**Affiliations:** 1 Nelson Mandela African Institute of Science and Technology, Arusha, Tanzania; 2 National Livestock Resources Research Institute, Tororo, Uganda; 3 Department of Global Health and Biomedical Sciences, Mzumbe University, Tanzania; 4 National Animal Disease Diagnostics and Epidemiology Centre, Entebbe, Uganda; 5 Institute of Biodiversity, Animal Health and Comparative Medicine, University of Glasgow, United Kingdom

## Abstract

Foot-and-mouth disease (FMD) is one of the major trans-boundary animal diseases in East Africa causing economic loss to farmers and other stakeholders in the livestock industry. Foot-and-mouth disease occurs widely in both Uganda and Tanzania with annual outbreaks recorded. With the recent introduction of the Progressive Control Pathway for FMD control (PCP-FMD) in eastern Africa, knowledge of the spatial and temporal distribution of FMD at the border area between Uganda and Tanzania is helpful in framing engagement with the initial stages of the PCP. Retrospective data collected between 2011 and 2016 from four districts located along the border areas of Uganda and Tanzania, recorded 23 and 59 FMD outbreaks, respectively, for the entire study period. Analysis showed that 46% of the 82 recorded outbreaks occurred in 20% of sub-counties and wards immediately neighbouring the Uganda–Tanzania border and 69.5% of the outbreaks occurred during the dry months. While the serotypes of the FMD virus responsible for most outbreaks reported in this region were not known, previous research reported South African Territory (SAT) 1, SAT 2 and O to be the serotypes in circulation. The results from this study provide evidence of the endemic status of FMD on the Uganda–Tanzania border and emphasise that the border area should be given due consideration during FMD control drives and that cross-border coordination should be prioritised. With the limited data on circulating serotypes in this area, there is a need for more vigilance on FMD case detection, laboratory diagnostic confirmation and provision of more complete documentation of outbreaks. This work further recommends more studies on cross-border livestock movement coupled with phylogenetics in order to understand the spread of the FMD in the border area.

## Introduction

Foot-and-mouth disease (FMD) is an endemic livestock disease in East Africa (Food and Agriculture Organization of the United Nations [FAO 2007]) caused by the foot-and-mouth disease virus (FMDV). The disease affects cattle, goats, sheep, pigs and other cloven hooved animals including wildlife. The virus is transmitted when susceptible animals are exposed to animals that have the virus. Although close proximity between animals may be necessary, some information suggests that the virus can be carried by the wind. Typically, the virus causes livestock to exhibit fevers; blisters in the mouth, tongue, hooves and udder; loss of appetite; anorexia; and excessive salivation. While the virus affects livestock of all ages, the young are most likely to die due to the disease, while older animals become less productive. Once the virus is active, it must run its course, which is why prevention of FMD is most effective.

Foot-and-mouth disease virus exists as seven serotypes: A, O, C, Asia 1, SAT 1, SAT 2 and SAT 3. Six of these serotypes (except for Asia 1) are known to have circulated in East Africa (Vosloo et al. 2002). Serotype O is the most predominant serotype and serotype A is widely distributed across East Africa. Among the SATs, SAT 2 is the most predominant, while SAT 3 is the least common. Serotype C has never been reported in Tanzania but was last reported in Uganda in 1971 and in Kenya in 2004. Since then, it has not been reported in East Africa (Sangula 2010).

Outbreaks of FMD occur widely throughout East Africa with cases recorded every year in parts of Uganda and Tanzania. Challenges remain with the implementation of control measures, such as vaccination and movement restriction, and thus FMD remains poorly controlled in both countries (Ministry of Agriculture Animal Industry and Fisheries [MAAIF] 2012; Vosloo et al. 2002). As a result, farmers, traders and national governments continue to incur direct and indirect costs associated with the disease annually (Baluka, Ocaido & Mugisha 2014; Kivaria 2003). Locally, FMD outbreaks affect the sale and purchase of livestock and livestock products, resulting in loss of income. Internationally, endemic countries are restricted from exporting their livestock and livestock products (James & Rushton 2002), resulting in loss of revenue. In addition, governments of affected countries may impose quarantine and vaccination campaigns, which are costly and may not be effective (James & Rushton 2002).

In recent years, growing attention has been given to understanding the epidemiology of FMD in Uganda and Tanzania (Ayebazibwe et al. 2010; Balinda et al. 2010; Genchwere & Kasanga 2014; Kasanga et al. 2011; Kivaria 2003; Namatovu et al. 2015). Research momentum has also been generated as a result of the introduction of the Progressive Control Pathway for Foot-and-Mouth Disease (PCP-FMD), a step-wise framework developed by the FAO and the World Organization for Animal Health (OIE) to assist FMD-endemic countries in reducing the impact of the disease (FAO 2011). The pathway in its initial stages requires the countries to have information on FMD outbreaks, including what serotypes are circulating, to understand the epidemiology of the disease and design strategic controls. Uganda and Tanzania are currently in the initial stages of the PCP-FMD, and both countries require accurate outbreak information to strategise the implementation of control measures (FAO 2011). However, information is still limited on the epidemiology of FMD, particularly at the border region between Uganda and Tanzania.

Border regions remain central in the epidemiology of trans-boundary animal diseases and these regions often suffer the burden of trans-boundary livestock diseases (Di Nardo, Knowles & Paton 2011). The uncontrolled movement of people and animals along borders has been documented as one of the major factors for the introduction and continued circulation of animal diseases, particularly FMD (Fèvre et al. 2006; Otte, Nugent & McLeod 2004). Between 2001 and 2008, sub-counties in Uganda that were adjacent to the Ugandan–Tanzanian border registered more outbreaks compared to the other Ugandan sub-counties situated along the Uganda–Democratic Republic of Congo border, Uganda–Kenya border, Uganda–Rwanda border and the Uganda–South Sudan border (Ayebazibwe et al. 2010). In addition, a study in Tanzania reported on the increased number of FMD outbreaks around Kagera (Picado et al. 2010), a Tanzanian region, which includes the Missenyi and Kyerwa districts and borders Uganda and Rwanda. Thus, the border area between Tanzania and Uganda remains an important area where more information on FMD outbreaks is helpful to inform strategies on regional control of the disease, which would be of great relevance and benefit to East African Community (EAC). Therefore, the objective of this study was to generate more information on spatial and temporal FMD outbreak patterns in the districts located along the border of Uganda and Tanzania.

## Materials and methods

### Study area

**FIGURE 1:**
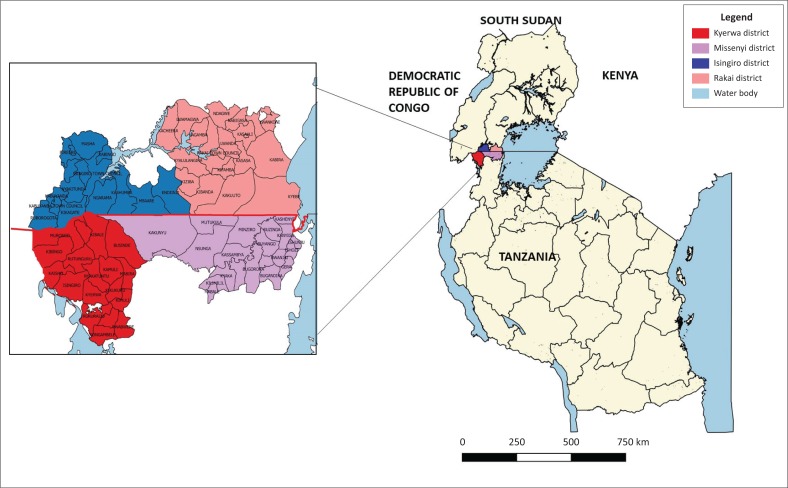
Study areas Isingiro and Rakai districts in Uganda and Missenyi and Kyerwa districts in Tanzania.

The study comprised of four districts located along the Uganda–Tanzania border. The study included two districts, Isingiro and Rakai, in Uganda located north of the Uganda–Tanzanian border, and the two districts of Missenyi and Kyerwa, in Tanzania located south of the Uganda–Tanzania border (Figure 1). These districts were purposively chosen because they share an international border. Isingiro and Rakai districts are composed of 10 and 20 sub-counties, respectively. In Uganda, a sub-county is the third lowest administrative level that is made up of several parishes, and within each parish is a number of villages. In Tanzania, the Missenyi district comprises of 20 wards, whereas the Kyerwa district has 18 wards. A ward in Tanzania serves as an administrative structure and is composed of several villages. The sub-counties and wards in Uganda and Tanzania are shown in Figure 1. In this study, sub-counties and wards that share an international border with either country will be addressed as adjacent or close to the international border.

### Data collection and management

Ugandan FMD outbreak records for the districts of Rakai and Isingiro were retrieved from the National Animal Disease Diagnostics and Epidemiology Centre (NADDEC) that carries out routine national surveillance in response to disease outbreaks. In the MAAIF (Uganda), the FMD reporting system works in such a way that FMD is considered as one of the notifiable diseases of public concern and has to be reported within 24 h to the commissioner of animal health (CAH). A suspected FMD case is reported by the affected farmer to the animal health staff (animal husbandry officer [AHO] or veterinary officer [VO]) in the sub-county who reports to the district veterinary officer [DVO]). The DVO establishes an interim quarantine and reports to the CAH by either an official letter or e-mail. The CAH dispatches a team to investigate the outbreak and confirm its presence or absence. The team collects appropriate samples which are analysed in the regional laboratories using mostly antibody and antigen enzyme-linked immunosorbent assay (ELISA) tests and occasionally polymerase chain reaction (PCR). After the confirmation of FMD, control measures such as quarantine and/or vaccination are instituted.

In Tanzania as well, FMD is considered a notifiable disease and must be reported within 24 hours. For the Tanzanian border districts of Missenyi and Kyerwa, retrospective data were retrieved from the records of the DVOs. In Tanzania, the system for reporting FMD is such that, a farmer reports to the livestock field officer (LFO) who goes to the affected farm to confirm the presence of FMD based on clinical signs. The LFO reports to the DVO. Ideally, appropriate samples are collected from the infected animals by the DVO and transported to the national laboratory (Tanzania Veterinary Laboratory Agency) for confirmation of FMD through antibody and antigen ELISA tests.

However, in this study, an FMD outbreak was defined as the presence of FMD clinical signs in at least one herd of cattle in a village within 1 month of the report of an outbreak. Most of the outbreak diagnoses were performed by the VOs in the districts, who based their diagnoses mainly on clinical signs exhibited by the affected animals. Cattle were considered as FMD-positive cases if they exhibited two or more of the following clinical signs – lesions in the mouth, on the gum, on the tongue, on the hooves and lesions on the mammary glands (in the case of females) accompanied by excessive salivation, fever, anorexia and lack of appetite.

For this study, data on serotypes that were in circulation during the study period were retrieved from the DVOs’ records as well as from NADDEC records. Other sources of this information were research works that had previously been carried out in this region (Genchwere & Kasanga 2014; Namatovu et al. 2015).

In the areas of this study, the seasons comprised wet and dry periods which are annually bimodal. The wet period consists of rainy days which occur in March and April and from September to November. The long dry season is characterised by periods of high temperatures averaging 27.5 °C – 30 °C and extends from December to February and from May to August (Isingiro District Local Government [IDLG] 2011; Rakai District Local Government 2013). The dry season in the Kagera region extends from December to February, and then from May to September, while the wet season is in March and April and then from October to December (FAO n.d.).

### Constraints of the study

Most of the data in this study consisted of reported cases based on clinical signs of FMD. A few samples were taken to the respective reference laboratories in the two countries for analysis to confirm the presence or absence of the disease by tests such as real-time or conventional PCR and antigen/antibody ELISAs, among others. Owing to lack of tests carried out for most of the reported cases, one cannot rule out that the symptoms could have been caused by other diseases that exhibit clinical signs similar to those of FMD such as bovine papular stomatitis, vesicular stomatitis, malignant catarrhal fever and bluetongue (Holliman 2005).

Other details were not included during data collection. Over 60% of the data lacked elements such as geographic positioning system (GPS) locations, number of cases registered during the outbreaks and number of animals at risk.

Cases in this study comprised only cattle because data were only collected for cattle. This study was based on retrospective data retrieved from monthly reports by the DVOs of the study districts and may be subject to underreporting by farmers owing to political, social and other reasons (Dhikusooka et al. 2015; Sutmoller et al. 2003).

### Data analysis

The data that were analysed comprised of the number of outbreaks in each sub-county and/or ward, the month in which the outbreak occurred, the district in which the sub-county/ward was located and whether the sub-county and/or ward was directly adjacent to the international border between Tanzania and Uganda. Data analysis was performed in R software, version 3.3.2 (R Core Team 2013), using generalised linear mixed effect models (package lme4) to describe the relationship between the response variable (number of outbreaks modelled as Poisson distribution) and the fixed (season, wet-dry, border adjacency [yes, no] and year of outbreak) and random (district, sub-county and/or ward, year) effects. Model selection was undertaken, and the significance of fixed effects was determined using likelihood ratio tests (LRTs).

**FIGURE 2:**
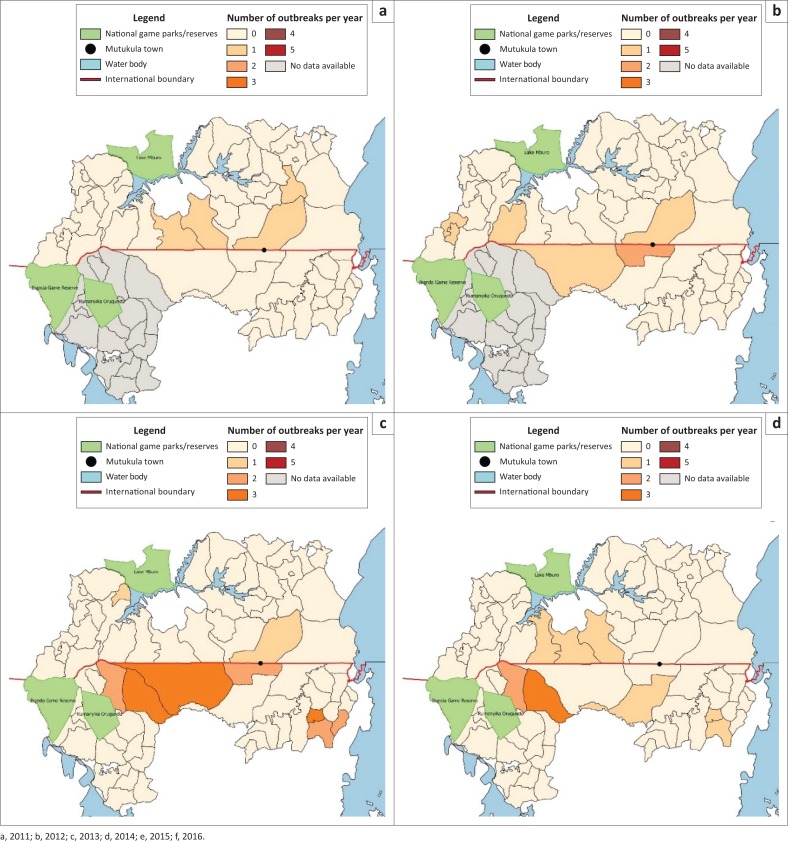

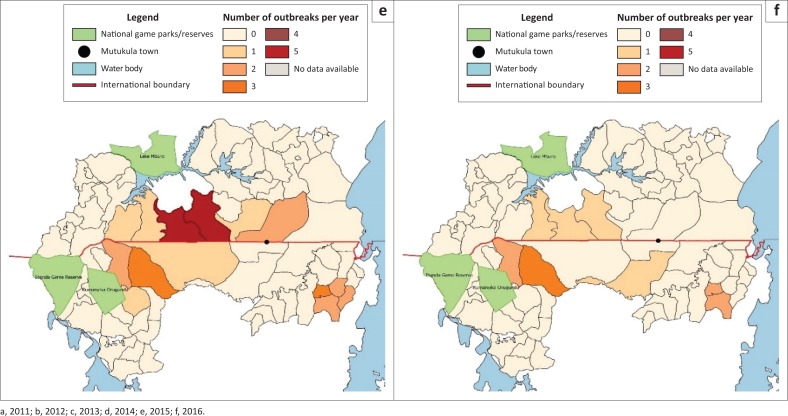
Spatial distribution of foot-and-mouth disease in sub-counties and/or wards in border districts of Uganda and Tanzania between 2011 and 2016.

Maps were prepared using QGIS 2.16.0 (Open Source Geospatial Foundation 2016) reflecting the sub-counties/wards where FMD outbreaks had occurred over the last 6 years. Because no GPS data on the exact location of affected villages were collected, analysis was conducted at the scale of sub-counties/wards where the affected villages were located (Figure 2).

## Results

### Spatial distribution

Between 2011 and 2016, a total of 23 outbreaks were reported to have occurred in the two Ugandan districts and 59 in the two Tanzanian districts located along the Uganda–Tanzania border. Throughout the entire period, the district of Missenyi in Tanzania had the highest number of outbreaks (36) followed by Kyerwa (23), Isingiro (15) and Rakai (8) (refer to Figure 2 on annual frequency trends). The latter two districts are in Uganda. Of the 82 outbreaks, 38 outbreaks (46%) occurred in sub-counties and/or wards that shared an international border with either country. Overall, the analysis showed that sub-counties/wards adjacent to the border reported three times more FMD outbreaks than the sub-counties/wards that were not adjacent to the border (*x*^2^ = 5.643, df = 1, *p* < 0.001).

### Temporal distribution of foot-and-mouth disease

**FIGURE 3:**
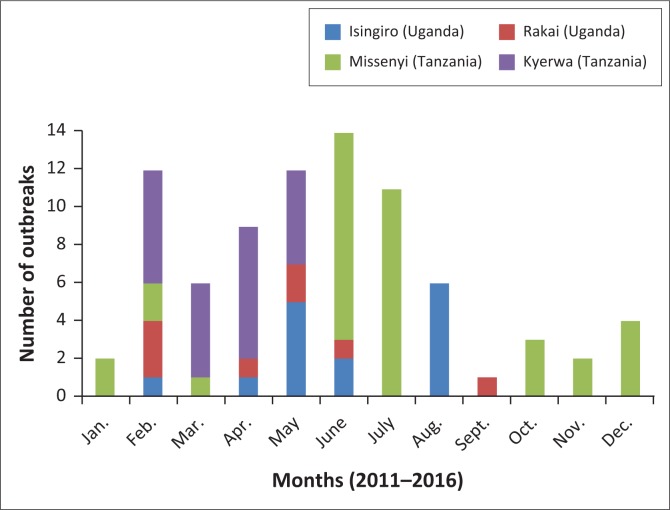
Temporal distribution of the number of outbreaks in selected border districts of Uganda and Tanzania.

Analysis of temporal data showed that the months with no outbreaks over the 6-year period in the two Ugandan border districts were January, March, July and October–December. Isingiro district reported the highest number of outbreaks to have occurred in August (6), whereas Rakai registered the most outbreaks in February (3) (Figure 3). In Missenyi district of Tanzania, most outbreaks occurred in June (10) and July (10), whereas in Kyerwa the highest number of outbreaks was reported in April (7). September was the only month with no outbreaks in both Tanzanian districts. The analysis showed that outbreaks were 2.7 times more frequent in the dry season than in the wet season (*x*^2^ = 18.311, df = 1, *p* < 0.001). Overall, the distribution of outbreaks was such that on average, outbreaks in the four districts occurred within an interval of three months apart; however, it is not clear if a pattern exists in the distribution of the reported outbreaks within the districts.

### Serotype distribution

Overall, from the data available on serotypes, three serotypes (O, SAT 1 and SAT 2) were found to be in circulation in the border region between 2011 and 2016 as shown in Table 1.

**TABLE 1:** Foot-and-mouth disease serotypes responsible for some of the outbreaks between 2011 and 2016 in the districts at the border of Uganda and Tanzania.

Year	Country	District	SC/ward	Serotype	Source
2011	Uganda	Rakai	Kasasa	O	Namatovu et al. 2015
2011	Tanzania	Missenyi	Mutukula	O	Genchwere and Kasanga, 2014
2013	Uganda	Isingiro	Kabingo	SAT 2	Namatovu et al. 2015
2015	Uganda	Isingiro	Endinzi	SAT 1	NADDEC
2015	Uganda	Isingiro	Kashumba	SAT 1	NADDEC
2015	Uganda	Isingiro	Ngarama	SAT 1	NADDEC

NADDEC, National Animal Diagnostic Disease and Epidemiology Centre; SC, sub-county.

## Discussion

The results showed that FMD outbreaks occurred in all four districts at the Uganda–Tanzania border over the period (2011–2016), reflecting the endemic nature of FMD in this border region. Overall, the study revealed that sub-counties and/or wards that were adjacent to the international border between Uganda and Tanzania were significantly more affected by FMD and most outbreaks occurred during dry season (Figures 2 and 3).

The high occurrence of outbreaks in Ugandan sub-counties adjacent to the border is consistent with findings by Di Nardo et al. (2011), who reported that border regions usually suffer the burden of trans-boundary livestock diseases fuelled by trade (both legal and illegal) between neighbouring countries. The increased number of outbreaks in this area is consistent with findings by Picado et al. (2010). In addition, Di Nardo et al. (2011) identified the trans-boundary area between Uganda and Tanzania as one of the main risk areas for FMD circulation in eastern Africa (Di Nardo et al. 2011). According to the Isingiro district local government, Isingiro district shares its border with Tanzania, which creates an opportunity for cross-border trade but also predisposes the district to an influx of pastoral immigrants from Tanzania who come with potentially infected animals, creating a constant potential source of diseases (IDLG 2011). So, while trade benefits border communities, it also creates or facilitates the trans-border spread of livestock diseases (Domenech et al. 2016). This produces a continual introduction or re-introduction of the disease from either side owing to uncontrolled human and animal migration (Di Nardo et al. 2011).

In the Missenyi district (Kakunyu ward), the same village of Bubale was consistently affected for 4 years, perhaps attributable to the location of a major livestock market in Bubale. Also in Uganda, the Kakuuto sub-county reported the most number of outbreaks in the Rakai district, while the sub-county of Endinzi reported the most in the Isingiro district. Both districts had recurrent outbreaks probably because of their major cattle markets located near the border. These finding are consistent with those that have shown that livestock markets are one of the major factors for FMD circulation and distribution in sub-Saharan Africa (Di Nardo et al. 2011).

Compared to the Ugandan districts, the Tanzanian districts had higher numbers of outbreaks. The two countries have different livestock disease policies for disease control. In Uganda, control of FMD is deemed a public good, with vaccines and vaccinations offered at a subsidised cost to farmers whenever available (Muleme et al. 2012). The situation in Tanzania is different. The National Livestock Policy by the Ministry of Livestock Development (MLD, 2006) does not mention governmental subsidisation of FMD vaccination costs. Therefore, farmers may be reluctant to vaccinate for FMD because of the full cost they must incur, which may contribute to the spread of the disease in the country. However, despite the imposed quarantines and regular vaccinations in the Ugandan district of Isingiro, FMD outbreaks are still frequent. This may be the result of resistance by some residents and leaders to imposed quarantines for economic and political reasons (Kabasongora 2014; New Vision 3 November 2016). Occasionally, in the Rakai district, some farmers take their animals into Tanzania to elude vaccination, citing vaccine ineffectiveness. They later return with their animals that may have been infected from Tanzania, complicating efforts in controlling FMD in Rakai (J. Kabagenyi [animal husbandry officer, Rakai district] pers. comm., 15 February 2016). Consequently, the continuous presence of FMD pockets on either side of the Uganda–Tanzania border increases the risk of FMD outbreaks in both countries, given that the outbreaks may spread beyond the border region.

In addition, wildlife is responsible for circulating SAT strains of the FMD virus, as has been studied in some parts of Africa (Jori & Etter 2016; Vosloo et al. 2005). However, it is not clear if the presence of wildlife is the reason for FMD persistence in the Kibale ward of the Kyerwa district. The Rumanyika Orugundu game reserve stretches into some parts of the Kamuli and the Kibale wards (Figure 2). The Kamuli ward reported only one outbreak during the entire 6-year period. Some studies have shown that areas located at wildlife–livestock interfaces have more FMD outbreaks than areas that do not harbour any wildlife (Ayebazibwe et al. 2010; Sinkala et al. 2014). Nevertheless, wards in Kyerwa, such as Murongo and Kibingo, that accommodate the Ibanda National Game Reserve did not register any outbreaks during the entire study period. In the Isingiro district, all the sub-counties located near Lake Mburo game reserve, save for Kabingo (in 2013), and did not report any outbreaks as well. It may appear that the presence of wild life may not be a major factor in FMD persistence in this border area. Further studies on risk factors for FMD persistence or new introductions in these areas are needed to determine the reasons behind persistent outbreaks.

Although Genchwere and Kasanga (2014) showed that most farmers in Tanzania were aware of FMD signs and farmers’ descriptions of FMD had a high correlation with positive ELISA results obtained from FMD outbreaks during their study, more reported cases should be confirmed by laboratory analysis. This would also address the dearth of information on FMD serotypes presented in outbreak report in both countries, mainly due to the limited laboratory and field capacity (Dhikusooka et al. 2015; Namatovu et al. 2013).

Muleme et al. (2012) reported on poor outbreak investigations and revealed that out of 121 outbreaks reported in Uganda between 2001 and 2010, the causal serotype was determined for only nine outbreaks. This further complicates any efforts for disease control given that circulating serotypes are unknown (Kitching et al. 2007). The presence of serotype O has recently been confirmed by other studies carried out in the Missenyi and Rakai districts (Genchwere & Kasanga 2014; Kerfua et al. 2013; Namatovu et al. 2015). Serotypes SAT 1 and SAT 2 have been previously detected by studies carried out in the Lake Zone region where both the Missenyi and Kyerwa districts are located (Swai, Mrosso & Masambu 2009). Also, the 2013 FMDV SAT 2 isolate from the Isingiro district in Uganda was closely related (97% similarity) to the VP 1 nucleotide coding sequences found in the isolate from Tanzania (TAN43/2009). This raises the question of whether the virus is spread between the two countries through trans-boundary movement of people and animals (Namatovu et al. 2015).

Most of the outbreaks occurred in the dry months of the year, consistent with previous findings (Ayebazibwe et al. 2010; Kivaria 2003). During the dry months, farmers move their animals for long distances in search of pasture and water and the interaction between animals at the water and pasture sources may facilitate easy spread of infectious diseases (VanderWaal et al. 2017). The patterns of outbreaks in all four districts also showed that the outbreaks occurred on average 3 months apart (data not shown), implying probable circulation of the virus between the districts. This argument may be inconclusive given that sequence and serotype data for this study were very limited.

## Conclusion

Areas adjacent to the borders and livestock markets may play a major role in FMD spread and thus FMD control can be strategised based on emphasis on such hot spots. Wildlife may not be as important in FMD spread in some areas and so further studies on risk factors have to be carried out for those areas that are persistently affected. This study underscores the need for better outbreak data documentation and the need for laboratory analysis and confirmation of FMDV serotypes during outbreaks reported in either country. It also recommends the need to determine genetic relatedness of FMDVs responsible for outbreaks in this region in order to improve our understanding of FMDV circulation and assist with its control. It is also imperative that neighbouring countries coordinate regional control strategies and enact and enforce laws regulating movement of livestock across borders for better management of FMD at border districts.
