# Corrigendum: Spatial and temporal distribution of foot-and-mouth disease in four districts situated along the Uganda–Tanzania border: Implications for cross-border efforts in disease control

**DOI:** 10.4102/ojvr.v85i1.1716

**Published:** 2018-12-11

**Authors:** Susan D. Kerfua, Gabriel Shirima, Lughano Kusiluka, Chrisostom Ayebazibwe, Robert Mwebe, Sarah Cleaveland, Daniel Haydon

**Affiliations:** 1 Nelson Mandela African Institute of Science and Technology, Arusha, Tanzania; 2 National Livestock Resources Research Institute, Tororo, Uganda; 3 Department of Global Health and Biomedical Sciences, Mzumbe University, Tanzania; 4 National Animal Disease Diagnostics and Epidemiology Centre, Entebbe, Uganda; 5 Institute of Biodiversity, Animal Health and Comparative Medicine, University of Glasgow, United Kingdom

In the author list of this article published earlier, Chrisostom Ayebazibwe’s name was unintentionally misprinted as ‘Chrisostome’. The correct name is ‘Chrisostom’. The author sincerely regrets this error and apologises for any inconvenience caused.

